# Digital pathology in forensic science: a systematic review of the literature

**DOI:** 10.1007/s12024-025-01146-w

**Published:** 2025-12-13

**Authors:** Laura Ambrosi, Federica Amirante, Simona Nicolì, Andrea Marzullo, Giuseppe Ingravallo, Davide Ferorelli, Biagio Solarino, Gianmarco Argentiero

**Affiliations:** 1 Interdisciplinary Department of Medicine, Section of Legal Medicine, University of Bari Aldo Moro, Piazza Giulio Cesare 11, Bari, Italy; 2 Department of Health Surveillance and Bioethics, Section of Legal Medicine, Fondazione Policlinico A. Gemelli IRCCS, Università Cattolica del Sacro Cuore, 00168 Rome, Italy; 3 Department of Precision and Regenerative Medicine and Ionian Area, Pathology Unit, University of Bari “Aldo Moro”, 70121 Bari, Italy; 4 Department of Public Health, Experimental and Forensic Medicine, Section of Legal Medicine and Forensic Sciences, University of Pavia, 27100 Pavia, Italy

**Keywords:** Forensic, Digital pathology, Whole slide image, Digital histopathology

## Abstract

**Background:**

Digital pathology (DP) and whole-slide imaging (WSI) are increasingly utilized in clinical pathology; however, their role in forensic medicine remains less defined, as evidentiary standards demand robust validation, auditability, and a chain of custody.

**Methods:**

We conducted a systematic review of PubMed, Scopus, and Web of Science for studies that applied DP and WSI to forensic, autopsy, or postmortem contexts, with eligibility requiring peer-reviewed human studies that reported methods and outcomes. Data were charted for study design, tissue, devices/software, and outcomes (diagnostic agreement, quantitative metrics, validation/quality assurance (QA)).

**Results:**

The search retrieved 361 records; after screening and full-text assessment, 21 studies were selected for inclusion. Fifteen studies primarily advanced diagnostic knowledge using postmortem material (e.g., quantitative neuropathology and organ-specific morphometry), while five had direct forensic aims (casework validation or core forensic tests).

**Conclusions:**

The review highlights that DP is technically ready for medico-legal workflows; however, its use remains low compared to other clinical settings. Adoption in forensics should centre on CAP-style, use–case–specific validation, traceable/auditable pipelines (including hashing, logs, and tile-linked overlays), stain/colour governance, and external robustness testing. Under these conditions, DP can deliver reproducible, transparent, and court-defensible evidence across forensic practice.

## Introduction

In recent years, digital pathology (DP) has undergone significant evolution, emerging as one of the most promising technological innovations in the field of diagnostic medicine [1]. DP involves the acquisition, management, sharing, and interpretation of pathology information, including slides and data, in a digital format.

Before the advent of high-throughput scanners and artificial intelligence (AI), digital pathology evolved from static imaging, particularly gross specimen photography, which was integrated into routine anatomic pathology and linked to reports and electronic medical records. Rampy and Glassy argue that high-quality gross photos are a core element of pathology practice, supporting case review, education, and medico-legal documentation when paired with appropriate metadata and storage [2].

Central to this process is whole-slide imaging (WSI), which enables high-resolution scanning of entire histological slides, transforming traditional microscopy into a digital workflow [3]. Initially, the first whole-slide scanners (WSS) were basic, taking a considerable amount of time to capture images and generating large files (approximately 7 Gigabytes of data for each focal plane) [4]. However, advancements in automation, scanner technology, file compression, high-quality lenses and objectives, improved camera sensors, and increased digital storage have significantly enhanced the usability and accessibility of WSIs, making them more attractive in the market [5].

The digitalization of slides allows for remote viewing, archiving, computational analysis and integration with AI systems. These capabilities have gradually reshaped pathology practices, with significant implications for clinical diagnostics, research, and education [6].

Historically, pathology image acquisition relied on static screenshots captured through optical microscopes, primarily used for documentation or teaching [6]. The introduction of telepathology in the 1980 s, which involved transmitting microscopic images via remote-controlled microscopes, marked a turning point. However, digital pathology only became scalable and practical for routine diagnosis once whole-slide imaging and strong IA infrastructure were available [7]. Today, WSI systems quickly digitize large sets of slides. AI-powered tools extract quantitative data from tissue samples, providing objective, reproducible measurements [8].

DP has shown significant advantages in clinical pathology. It promotes standardization, enhances collaborative diagnostics, facilitates remote consultations, and supports AI-driven image analysis. Additionally, it allows for retrospective analysis and validation of results [9]. AI and machine learning algorithms are now utilized to identify tumor margins, categorize tissue types, assess biomarker expression, and predict molecular features based on morphological patterns. These tools not only enhance diagnostic accuracy but also reduce inter-observer variability and expedite turnaround times (TAT), resulting in improved patient outcomes [10]. Specifically, computational pathology (CPATH) has risen as a subfield of DP, integrating WSI, big data analytics, and artificial intelligence (AI) to support precision medicine [6].

Nowadays, pathology diagnosis using WSI on computer monitor screens has been approved by the Food and Drug Administration of the United States and worldwide [11–13].

Despite the widespread adoption of digital pathology in medical fields such as oncology, molecular pathology, and toxicologic pathology, its use in forensic settings remains surprisingly limited. Forensic pathology encompasses a wide range of case types, many of which require careful documentation, reproducibility, and objectivity, features that digital systems are well-suited to provide.

The potential applications of DP in forensic pathology are significant and largely underexplored.

As a proof of concept, Porzionato et al. demonstrated the value of digital 3D reconstruction in forensic histopathology by virtually reconstructing the whole microscopic trajectory of a cardiac stab wound. Using serial histological sections and open-source image processing software, the authors generated a three-dimensional model of the lesion, enabling detailed morphometric analysis and correlation with the suspected knife [14].

Moreover, digital image analysis can offer objective measurement of wound areas and aid in pattern recognition (such as firearm injuries, healing burns, and patterned contusions). A promising technique, Spatially Invariant Vector Quantization (SIVQ), has been demonstrated to enable non-programmer forensic pathologists to perform advanced pattern detection with simple point-and-click actions on gross images, yielding both qualitative and quantitative results [15].

Furthermore, DP can enhance documentation and chain-of-custody management in forensic work. Digital archiving supports the secure and traceable storage of histological data, reduces the risk of slide degradation or loss, and enables the re-examination of materials by external experts even years later. In high-profile or controversial cases, this ability can be crucial in ensuring the transparency and reproducibility of forensic diagnoses [16].

Despite the promise, current studies on DP in forensics are limited and vary widely in methods. Most validation research focuses on clinical specialties, such as prostate, breast, lung, and gastrointestinal pathology, and often faces problems including narrow case selection, small sample sizes, and a lack of intraobserver reliability testing [17–20]. Very few studies cover the entire spectrum of tissue types and diagnostic challenges faced in forensic pathology.

However, several barriers have blocked widespread adoption in this area. These challenges include the absence of standardized validation procedures and the perceived legal uncertainty around the admissibility of digital images as evidence in court. As noted by the Royal College of Pathologists, digital images, while visually attractive, may lack diagnostic details if poorly captured, and the same tissue sample can appear differently depending on the scanning equipment and software used. Most validation studies focus on clinical subspecialties and often have limitations, such as limited case selection, small sample sizes, and a lack of intraobserver consistency testing [7]. Additionally, questions remain about data storage, long-term archiving, and who is responsible for system failures or misinterpretations in legal situations.

Very few studies cover the full range of tissue types and diagnostic challenges faced in forensic pathology. To realize the significant potential of digital pathology for practical use in forensics, there is an urgent need for thorough validation studies and the development of standardized protocols that meet the specific operational, diagnostic, and legal requirements of forensic work.

Considering these gaps in the literature, a critical examination of the current role and further prospects of DP in forensic medicine is necessary. As emphasized by recent guidelines, digital systems must undergo thorough, context-specific validation before being used for primary diagnosis. Validation should be viewed not as a one-time process, but as an ongoing learning journey, involving feedback loops, training, and the transparent assessment of the system's strengths and limitations [7].

The primary aim of this review is to provide a comprehensive and current overview of the role of digital pathology (DP) in modern medical and forensic practice. Although the use of DP in forensic settings is still limited, its potential spans various aspects of forensic pathology, including quantifying wound patterns, standardizing histological assessments, enhancing archiving techniques, and facilitating expert consultations and legal reviews. This review will examine not only the technical feasibility of these applications but also their medico-legal and operational impacts, highlighting specific areas where digital pathology could significantly improve forensic workflows. Additionally, the review aims to offer a critical, evidence-based perspective on the challenges, limitations, and conditions necessary for broader integration of digital pathology into forensic work. This analysis encompasses existing validation studies, infrastructure requirements, and legal considerations, as well as identifying research gaps and outlining future directions.

## Materials and methods

This systematic review adhered to the Preferred Reporting Items for Systematic Review and Meta-Analyses (PRISMA) guidelines [21].

### Search strategy

A comprehensive literature search was conducted across three major databases: PubMed, Scopus, and Web of Science. The search strategy combined terms related to digital pathology (e.g., "digital patholog*", "digital histolog*", "whole slide image*", "digital histopatholog*") and the field of its application (e.g., "forensic*", "forensic science*", "autops*", "criminalistic*", "legal medicine", "postmortem", "postmortem"). Boolean operators and wildcards were used to ensure broad retrieval. The final search was performed on 11th June 2025.

Three investigators (GA, LA, and SN) independently conducted the selection process and extracted data, following PRISMA standards, with two repetitions to minimize study bias. Three investigators (GA, LA, and FA) verified the data, and two other investigators (AM and DF) validated the methodology for the qualitative synthesis. The final work was revised by an external investigator as supervisor (BS). An additional external expert (GI) performed a further scientific and linguistic revision of the manuscript.

### Inclusion and exclusion criteria

Articles were eligible if they were: (1) published in peer-reviewed journals; (2) focused on applying digital pathology, digital histology, whole-slide imaging, or image analysis to forensic, autopsy, postmortem samples, or legal medicine contexts; (3) published in English; (4) provided sufficient methodological details and reported quantitative or qualitative outcomes related to digital pathology applications. The inclusion criteria did not impose any restriction on the year of publication.

The authors excluded review articles, conference abstracts, editorial letters, studies unrelated to forensic or autopsy settings, and studies that did not involve human subjects. Grey literature and preprints were excluded to ensure that only peer-reviewed and methodologically validated studies were included in the analysis.

### Study selection

**Fig. 1:**
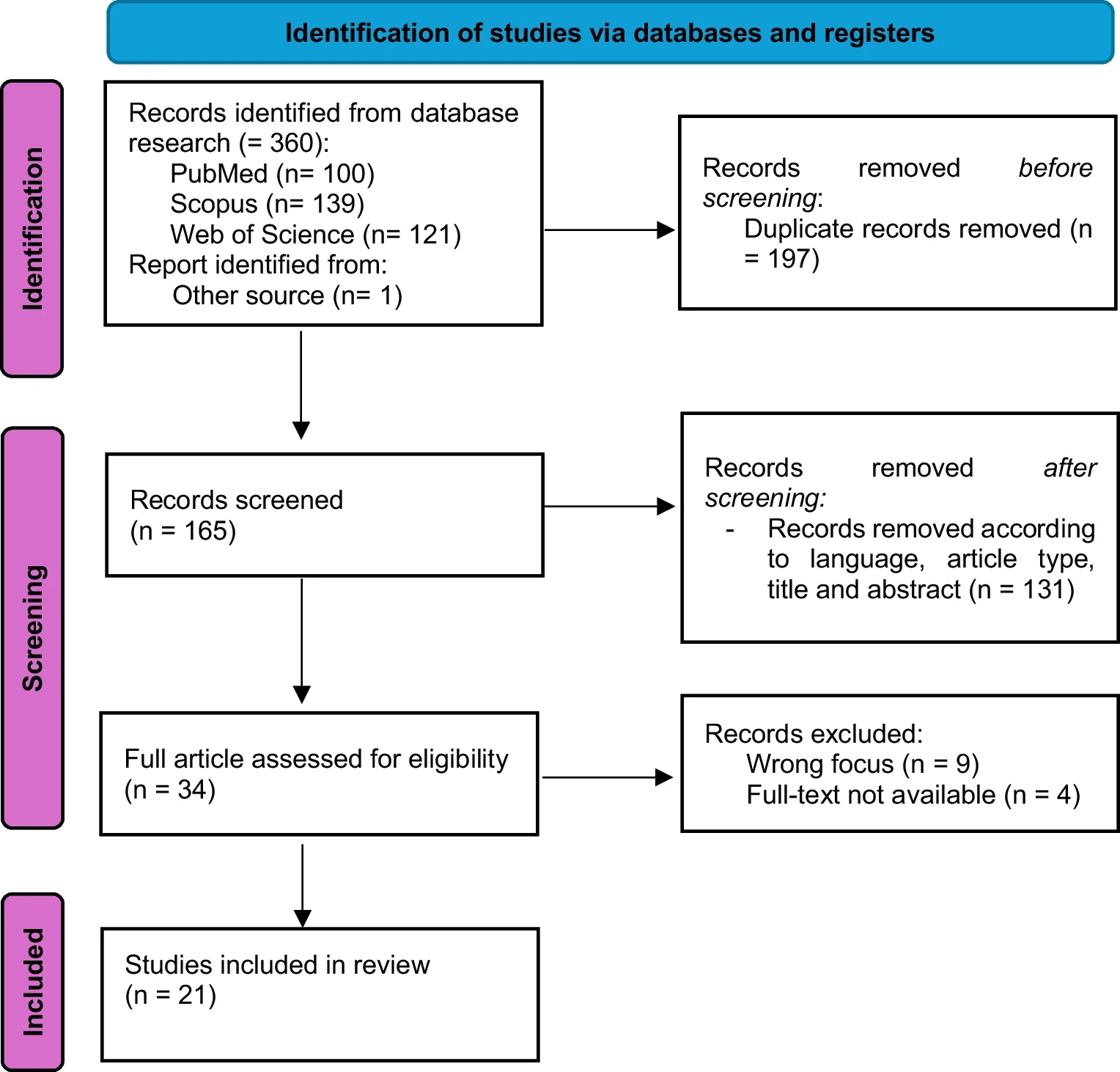
PRISMA 2020 flow diagram illustrating the study selection process

The initial search retrieved a total of 360 articles published between 2009 and 2025. One article was recovered from another source. After removing duplicates, 165 unique records remained. A two-step screening process was applied. Screening of titles and abstracts excluded 131 articles due to article types and incorrect focus. Full-text versions of the remaining 34 articles were assessed for eligibility. Four articles were excluded because their full texts were unavailable, leaving 30 articles for detailed evaluation. After full-text evaluation, conducted independently by two reviewers in duplicate, 21 articles met the inclusion criteria and were included in the final review. Discrepancies were resolved by consulting a third reviewer or, when necessary, through Delphi rounds with all authors. We present the PRISMA flowchart in Fig. 1.

### Data extraction

Three reviewers independently extracted data in duplicate using a standardized data-charting form to aid in assessing study quality and synthesizing evidence. The following information was recorded for each included study: (1) First author, publication year, journal; (2) Study type and location; (3) Population characteristics and sample type; (4) Staining methods and use of immunohistochemistry; (5) Device and software used; (6) Main outcomes.

### Quality assessment

Two reviewers independently conducted a quality assessment to evaluate the quality of the selected studies. The methodological quality of the included studies was assessed using the appropriate Joanna Briggs Institute (JBI) Critical Appraisal Tools, chosen based on the specific study design (e.g., case–control, cross-sectional, quasi-experimental) [22]. The tools consisted of eight to eleven questions, each answerable with "yes," "no," "unclear," or "not applicable," along with a final "overall appraisal" judgment that includes the options: "Include", "Exclude", and "Seek further information". The process also allows for a brief comment to justify any exclusion.

## Results

Among the 21 studies included, publication years range from 2009 to 2025 and cover various regions in Europe, North America, and Asia. The study designs are mainly observational (retrospective or cross-sectional), with a smaller number of methodological or pilot studies; sample sizes vary from small technical series to larger cohorts. Table 1 summarizes the study characteristics and main findings.

**Table 1:** Characteristics of the selected papers

Author (Year)	Study Location	Title	Study Type	Population	Sample Type	Staining Method	IHC	Device	Software/Program	Main Outcome and Forensic Relevance
D'Abbronzo et al (2024)	Italy	Application of Digital Analysis for Assessment of Coronary Sub-Occlusions in Autopsy Pathology: It Is Time to Move beyond Histology Alone	Original research	11 autopsies	Coronary artery cross-sections	H&E	No	Ventana DP2000 Slide Scanner	QuPath v. 4–3	Digital morphometry of coronary lumen gives objective %-stenosis; threshold for critical occlusion identified
Gheban et al (2022)	Romania	Techniques for digital histological morphometry of the pineal gland	Technical protocol	72 pineal glands from adult autopsy cases	Pineal glands	H&E	Yes	Pannoramic SCAN 150	CaseViewer by 3DHISTECH; ImageJ/Fiji; MorphoRGB	Established a reproducible digital morphometry protocol for pineal gland analysis using open-source software
Brunner et al (2022)	Austria	Visible and Near-Infrared hyperspectral imaging (HSI) can reliably quantify CD3 and CD45 positive inflammatory cells in myocarditis: Pilot study on formalin-fixed paraffin-embedded specimens from myocard obtained during autopsy	Pilot Study	40 autopsy patients with suspected myocarditis	Myocardial tissue	H&E	Yes	SNAPSCAN camera	MatLab 2021b	HSI reliably quantifies CD3 + and CD45 + cells in myocarditis with high concordance to visual assessment and digital image analysis
Cooze et al (2022)	UK-Italy	The association between neurodegeneration and local complement activation in the thalamus to progressive multiple sclerosis outcome	Observational Study	Tissue from 21 MS, 10 non neurological control cases, 4 inflammatory disease (viral encephalitis), 3 ischaemic encephalopathy from Tissue Bank and Oxford Bank	Brain	/	Yes	ZEISS Axio Scanner	Qupath	Complement activation (C1q, C3d) is associated with axonal loss and microglial activation in the thalamus of MS patients
Ichimata et al (2023)	Canada	The molecular spectrum of amyloid-beta (AB) in neurodegenerative disease beyond Alzheimer's disease	Observational Study	116 brain donations (51 LBD, 10 MSA, 39 PSP, 16 FTLD-TDP)	Brain	Hematoxylin	Yes	TissueScope LE120 slide scanner	ImageJ/Fiji	Different Aβ variants are present in non-AD neurodegenerative diseases, suggesting disease-specific Aβ profiles
Kinoshita et al (2017)	Japan	Proliferation of elastic fibres in idiopathic pulmonary fibrosis: a whole-slide image analysis and comparison with pleuroparenchymal fibroelastosis	Quantitative image analysis study	48 autopsies and pneuumonectomy patients	Lungs	H&E, EVG, Masson's trichrome	No	NanoZoomer 2.0-RS	ImageJ 1.49v	IPF occasionally shows intense elastosis in the upper lobes, and such cases are histologically indistinguishable from PPFE
Akyildiz et al (2009)	Turkey	Computerized Image Analysis in Differentiation of Skin Lesions Caused by Electrocution, Flame Burns, and Abrasion	Observational Study	30 deaths by electrocution, 30 deaths by flame burns, 30 deaths from traffic accident	Skin lesions	H&E	No	Nikon Eclipse 80i microscope	SAMBA 4000	Significant morphometric differences in nuclear dimensions help differentiate skin lesions caused by electrocution, flame burns, and abrasions
Kim et al (2025)	USA	Reliability and modeling of digital histological measurements in Alzheimer's disease neuropathologic change and Lewy body disease	Experimental study	24 autopsies	Brain	Hematoxylin	Yes	Zeiss AxioScan Z1	Qupath	Digital histological quantification (% area occupied by p-Tau, Aβ, and α-synuclein) demonstrated strong reproducibility and provided continuous, anatomically-resolved measures that correlated with neuropathological staging and cognitive performance
Ravi et al (2019)	India	Regional variation of human pancreatic islets dimension and its impact on beta cells in Indian population	Observational study	33 autopsies patient non-diabetic	Pancreas	H&E	Yes	Carl Zeiss digital slide scanning system	Fiji/ImageJ free software	Islet size and β-cell density varied significantly across pancreatic regions, with larger islets and higher β-cell proportion in the tail. These regional differences have implications for diabetes research and β-cell quantification
Crist et al (2021)	USA	Transcriptomic analysis to identify genes associated with selective hippocampal vulnerability in Alzheimer's disease	Observational study	40 postmortem human brains with AD and 15 controls	Brain	/	Yes	NanoString nCounter platform	MAP-Rseq v.20; TopHat v.2.0	Identification of SERPINA5, RYBP, SLC38A2, FEM1B, and PYDC1 as genes associated with hippocampal vulnerability in Alzheimer’s disease
Gheban et al (2022)	Romania	Digital histological morphometry of the human pineal gland in a postmortem study, with endocrine and neurological clinical implications	Observational study	72 autopsy cases of paediatric and adult patients	Pineal Glands	H&E	Yes	3D HISTECH PANNORAMIC SCAN II	3D HISTECH software Case-Viewer; Photoshop; Fiji	These findings provide morpho-functional insight regarding the role of the pineal gland in different pathologies and suggest that the pineal gland should not be neglected in standard clinical, imagistic, and autopsy evaluation
Nelther et al (2013)	USA	Arteriolosclerosis that affects multiple brain regions is linked to hippocampal sclerosis of ageing	Observational study	39 autopsy cases with HS-ageing pathology and 288 autopsy cases without HS-ageing	Brain	H&E	Yes	Aperio ScanScope XT	Aperio Microvessel Analysis algorithm	Found strong association between arteriolosclerosis in multiple brain regions and hippocampal sclerosis of aging, independent of TDP-43 pathology
Zhou et al (2019)	China	Digital whole-slide imaging analysis for automated diatom test in forensic cases of drowning using a convolutional neural nework algorithm	Experimental study	Lungs tissue from 15 non-drowning cases and 5 drowning cases	Diatoms	/	No	Leica, Aperio-AT2	GoogleNet Inception V3 arichtecture	Demonstrated high sensitivity and specificity of CNN-based automated diatom detection, supporting its forensic utility
Sobin et al (2024)	USA	Histologic and Quality Assessment of Genotype-Tissue Expression (GTEx) Research Samples	Observational Study	956 (postmortem donors and organ donors)	Different type of tissues	H&E	No	Aperio Scanscope software	/	95% of tissue samples were suitable for RNA sequencing. RNA integrity and autolysis varied by tissue type. Postmortem interval and agonal phase affected RNA quality in a tissue-specific manner
Benevento et al (2025)	Italy-Poland	Dura mater and survival time determination in individuals who died after traumatic brain injury: a preliminary study	Observational study	36 autposy cases (20 traumatic brain injury, 16 case control)	Dura mater	H&E	Yes	Not reported	QuPath	WSI + IHC on dura yields an objective, auditable metric for survival-time estimation after TBI: CD15 aligns with very-early survival, CD3 with day-scale survival; diagnostic signal preserved with mild putrefaction
Pigaiani et al (2025)	Italy-USA	Forensic, multicentric validation of digital whole slide images (WSI) in forensic histopathology setting according to the College of American Pathologists guidelines	Prospective study	100 histopathological glass slides (10 homicide, 15 suicide, 30 natural, 15 accidental, 30 medical liability)	Different type of tissues	H&E, Masson, Trichrome, Perls Von Kossa, PAS	Yes	Aperio GT 450 DX Slides Scanner	/	Diagnostic concordance between glass slides and WSI was 97.8%; CSUQ satisfaction score was 6.6/7. WSI deemed suitable for routine forensic pathology applications
Corredor et al (2021)	USA	Computational pathology reveals unique spatial patterns of immune response in H&E images from COVID-19 autopsies: preliminary findings	Observational Study	11 patients died due to H1N1 or COVID-19	Lungs	H&E	No	Ventana iScan HT Scanner; Aperio AT Turbo	/	COVID-19 lung samples exhibited multiple small lymphocyte clusters with limited immune activity, whereas H1N1 cases demonstrated larger, spatially cohesive lymphocyte and non-lymphocyte clusters, indicating a more robust and coordinated immune response
Hainsworth et al (2017)	UK	Neuropathology of white matter lesions, blood–brain barrier dysunction and dementia	Prospective longitudinal study	126 donated brain samples over 65 y.o	Brain	H&E	Yes	Leica SlideScanner	NIH ImageJ free software	Our data suggest that some degree of BBB dysfunction is common in older people and that this may be related to clinical dementia risk, additional to standard MRI biomarkers
Kapasi et al (2023)	USA	High-throughput digital quantification of Alzheimer disease pathology and associated infrastructure in large autopsy studies	Retrospective study	Older adults from 5 cohort studies (ROS, MAP, MARS, LATC, AA Core)	Brain	/	Yes	Aperio AT2 scanner	eSlideManager, ImageScope, Aperio Image Analysis Toolbox	Digital quantification of β-amyloid and tau-tangles is robust, reproducible, and harmonizable with historical stereological data. Digital pathology offers multiple advantages that overcomes critical challenges to traditional microscopy methods
Oliveira et al (2024)	USA	A machine learning approach to automate microinfarct and microhemorrhage screening in hematoxylin and eosin-stained human brain tissues	Experimental study	22 cases from brain bank with AD	Brain	H&E	No	Leica Aperio AT2; Zeiss Axio Z1 scanner	Imagescope; ZEN 3.0 blue edition	Deep-learning digital pathology accurately identified cerebrovascular microlesions on H&E-stained slides, achieving 100% sensitivity and specificity. Multi-resolution models outperformed single-scale approaches for lesions ≥ 1 mm. Results support its use for automated screening in neuropathology
Li et al (2024)	China	A diagnostic strategy for pulmonary fat embolism based on routine H&E staning using computational pathology	Observational – Retrospective Study	100 autopsy cases (traffic accident, blunt trauma, falls)	Lungs	H&E; oil red O-stained	No	Aperio AT2 Leica	GoogleNet-InceptionV3	Digital pathology enabled standardized quantification of pulmonary artery occlusion in fatal pulmonary embolism cases. The method demonstrated consistency across observers and highlighted its value in supporting forensic diagnosis of PE

The forensic settings examined include postmortem histology (notably of the brain, lung, heart, and skin), documentation of patterned injuries, and computational analyses based on WSI. Fifteen studies utilize postmortem samples primarily to enhance diagnostic knowledge, such as neuropathology pipelines and organ-specific histology. In comparison, six studies have a direct forensic focus, including validation for casework or tests targeting core forensic diagnoses.

For applications using postmortem samples to enhance diagnostic knowledge, Kapasi et al. operationalised a genuinely high-throughput Alzheimer's pipeline built on WSI-first processing across five brain-ageing cohorts [23]. Glass slides were digitised on a Leica/Aperio platform (AT-series) and managed in eSlideManager/ImageScope, with batch image analysis routines applied to pre-defined neuroanatomical regions. The authors report three reproducibility checks embedded in the workflow: repeat scans of the same slide, cross-magnification consistency (20 × vs 40 ×), and re-analysis of exported WSIs after storage/retrieval, each showing stable readouts. Quantitatively, digital burden estimates for β-amyloid and tau were highly correlated with historical manual measures **(**r = 0.83–0.94**)** and maintained that level of agreement across magnifications and rescans, indicating that throughput gains did not come at the expense of measurement fidelity. In addition, because the study pooled material from five independent cohorts, the team implemented a basic harmonization step (standard staining/scan parameters, as well as shared analysis presets) to ensure that outputs remained comparable at scale.

Oliveira et al. implemented a patch-based convolutional neural network (CNN) pipeline, in which WSI of postmortem haematoxylin and eosin (H&E) brain tissue were tiled into small image patches for patch-level classification and then re-aggregated to produce slide-level screening outputs and lesion heatmaps [24]. The dataset comprised 22 autopsy cases (66 slides: 40 WSIs with and 26 without microinfarcts/microhaemorrhages), sampled from periventricular white matter regions prone to microlesions. WSIs were digitised on Leica Aperio AT2 (SVS) and ZEISS Axio Scan Z1 (CZI), with expert annotations created in ImageScope and ZEN.

Beyond these flagship studies, the diagnostic process also involves targeted, organ-specific contributions that demonstrate how DP stabilizes measurement in postmortem tissues. Gheban et al. described a practical workflow for the pineal gland, using digitized sections analyzed with open-source tools to standardize region selection, morphometric measurements, and reporting, thus reducing operator-dependent variability in small neuroendocrine structures [25]. Gheban et al., in another study, expanded on this by performing morpho-functional analyses that combined quantitative histology on WSIs with structured descriptors of pineal parenchyma and stroma, offering a reproducible template for case-to-case comparisons [26]. Meanwhile, exemplars of vascular and neurodegenerative research, such as Hainsworth et al. on blood–brain barrier dysfunction in aging brains and Neltner et al. on TAR DNA binding protein 43 (TDP-43) pathology in hippocampal sclerosis of aging, demonstrate how digitized slides and quantitative annotations facilitate consistent lesion mapping and severity scoring across cohorts, strengthening the connection between postmortem histology and meaningful clinical neuropathological constructs [27, 28].

At the tissue-resource level, Sobin et al. evaluated ~ 30,000 postmortem specimens within the GTEx programme using a standardized digital quality control (QC) workflow, in which board-certified pathologists reviewed H&E slides to verify tissue identity and score pre-analytical quality (notably autolysis and section artifacts) alongside molecular suitability indicators [29]. Despite expected variability in autolysis and RNA integrity across organs and postmortem intervals, 95% of samples met the programme's thresholds for downstream RNA-seq. The remaining fraction was predominantly excluded due to severe autolysis or sub-threshold RNA quality, with exclusion rates varying by tissue type.

For applications with direct forensic value, Pigaiani et al. conducted a multicentre, CAP (College of American Pathologists)-aligned equivalence/non-inferiority validation in which six forensic pathologists first rendered diagnoses on 100 glass slides (H&E and special stains) and, after a washout, re-read the digitised counterparts scanned on a Leica/Aperio GT-450 DX and reviewed with the O3 Viewer [4]. The primary endpoint, diagnostic concordance (glass vs WSI), was 97.8% overall; concordance was high across both H&E and special stains. Secondary, operational readouts documented a mean scan time ≈ of approximately 44 s per slide and usability on the Computer System Usability Questionnaire **(**CSUQ), with a mean score of 6.6/7. The study standardised the reading workflow (blinded crossover with washout), reported site-pooled outcomes, and catalogued the small number of discordant calls following the CAP schema, thereby providing a comprehensive, reader-level, and system-level performance snapshot for routine forensic histopathology under WSI.

Li et al. evaluated a computational H&E-only strategy for pulmonary fat embolism (PFE) by digitising routine lung sections on an Aperio AT2 and training an Inception-V3 (GoogLeNet) pipeline to detect intravascular fat droplets on tiles and aggregate tile-level probabilities into a slide/case-level fat-area (%) readout [30]. Expert-annotated WSIs served as the ground truth for droplet localization, with performance at the droplet level reaching an Area Under the ROC Curve (AUC) of 0.98. Using the continuous fat-area output as a surrogate burden measure, the authors derived a data-driven decision threshold of 8.275% (optimized on the study set), which separated fatal PFE from controls with an AUC of 0.984 at the case level. The paper also benchmarked the digital readout against established references, including Oil Red O (lipid stain) and Falzi scoring, and reported close agreement while avoiding special staining. Operational details include WSI-scale inference with tile aggregation, and heatmap overlays to visualise detections in large vessels and capillary beds. The authors document sensitivity to acquisition parameters (scanner settings, colour characteristics), emphasizing that model performance can drift if slide digitization changes, and recommend calibration/maintenance as part of deployment. Overall, the results demonstrate that a scanner-agnostic, H&E-based workflow can yield a quantitative fat-area metric that aligns with reference methods and supports accurate PFE classification in an autopsy setting.

Zhou et al. implemented an end-to-end, CNN-assisted diatom test that mirrors the forensic workflow, replacing the most labor-intensive steps with automated image analysis [31]. After acid digestion and smear preparation, slides were scanned as WSIs on a Leica Aperio AT2 (typically 40 ×) and partitioned into tiles for Inception-V3 classification. The system produced two readouts in parallel: tile-level detections of diatom frustules, accompanied by Grad-CAM heatmaps, to verify that the network's attention coincided with the siliceous structures of interest, and slide-level counts aggregated from positive tiles, emulating the expert enumeration process. In validation, AI-assisted counts matched expert results, while total processing time fell from ~ 6–7 h (manual) to ~ 4 h with the automated pipeline. Classification performance was high, with an accuracy of ~ 97.7% and an AUC of ~ 99.5%. Error analysis revealed that most false positives originated from mineral/particulate debris morphologically similar to diatoms, a known challenge in digested smears. By coupling WSI-scale screening with saliency-based verification, the pipeline preserved traceability of each detection. It delivered a practical throughput gain for drowning investigations, including heavily decomposed cases where the diatom test is often pivotal.

D'Abbronzo et al. digitised coronary cross-sections from autopsies and applied a planimetric WSI workflow in which the luminal boundary and the internal elastic lamina (IEL) were delineated on H&E sections; the software then computed lumen and vessel areas to derive percentage area stenosis [32]. Using this quantitative readout, the study demonstrated tight repeatability and inter-rater consistency of measurements, as well as a close alignment with routine, semi-quantitative glass-slide grading. Notably, in borderline "sub-occlusion" cases, the numeric output reduced ambiguity by placing lesions on a continuous scale rather than broad categorical bins, thereby clarifying attribution in suspected ischaemic deaths. As each estimate is tied to the underlying WSI, which stores contours and measurement logs, the approach also yields an auditable trail (snapshots and metrics) that can be revisited alongside the histology during case reviews and expert testimony.

Benevento et al. digitised 108 immunohistochemical dura mater slides from 36 autopsies (20 with traumatic brain injuries; 16 controls) and used QuPath to establish objective, ROC-based thresholds based on positive-cell percentages [33]. CD15 immunoreactivity distinguished immediate death from survival lasting minutes or hours, while CD3 helped identify survival over days. Notably, diagnostic accuracy was maintained in mildly putrefied cases. These findings demonstrate how WSI-assisted cell detection can turn vitality and timing markers into reproducible, auditable metrics suitable for forensic workflows.

Finally, Corredor et al. analysed autopsy lung WSIs from 9 COVID-19 and Influenza A (H1N1) virus fatalities to quantify the spatial architecture of immune cells directly on routine H&E [34]. Slides were digitized at 20 × (NIH) or 40 × (Cleveland Clinic, University Hospitals, OCME); they were then harmonized to 20 ×. The scanners explicitly reported were Ventana iScan HT (CC, UH) and Aperio AT Turbo** (**OCME). Results showed that COVID-19 lungs consistently harbored numerous small, tightly packed lymphocyte clusters that frequently overlapped with non-lymphocyte clusters, whereas H1N1 lungs exhibited fewer but larger lymphocyte clusters adjacent to huge non-lymphocyte clusters. Heatmaps corroborated a higher non-lymphocyte density in H1N1 lungs. The study thereby provides a slide-native, quantitative description of immune-cell organisation, distinguishing COVID-19 from H1N1 using routine H&E WSI.

Across the set, hardware and software choices were broadly consistent, yet variably documented to a level suitable for replication. Leica/Aperio platforms predominated, most commonly AT2/AT Turbo for research workflows and GT-450 DX in the multicentre validation, alongside 3DHISTECH PANNORAMIC (pineal series) and ZEISS systems (e.g., Axio/Zen in neuropathology pipelines). Viewers and analysis tools clustered around ImageScope/eSlideManager (Aperio ecosystems), CaseViewer (3DHISTECH), and open-source stacks such as QuPath** a**nd Fiji/ImageJ; several pipelines combined vendor viewers for annotation with open-source or custom scripts for quantification. File containers reflected the vendor mix (SVS**,** CZI), with occasional scanner-agnostic handling explicitly shown when the same model was run on slides from different devices (e.g., Aperio + ZEISS). Where specified, slides were scanned at 20 × and/or 40 × (pixel sizes ~ 0.5–0.25 µm/px). The only formal throughput metric reported was in the study of Pigaiani et al. **(**~ 44 s/slide under routine conditions) [4]. Staining was predominantly H&E, with targeted immunohistochemistry (IHC) applied for specific diagnostic questions. Few papers detailed colour/stain normalisation or scanner calibration protocols, although some reported reproducibility checks across magnifications, rescans, or sites (e.g., repeat-scan consistency, cross-magnification stability, and harmonisation to a standard magnification before analysis).

In terms of validation reporting, the multicentre WSI study by Pigaiani et al. explicitly followed CAP-aligned elements and provided both diagnostic concordance (97.8%) and usability (CSUQ 6.6/7; mean scan time ≈44 s/slide), offering the most precise system-level snapshot among the included works [4]. By contrast, AI-oriented pipelines chiefly reported discrimination metrics on internally split datasets, often with saliency overlays for traceability, for example, Li et al. on pulmonary fat embolism (AUC 0.98 for droplet detection [30]; Zhou et al. on the diatom test (accuracy ≈97.7%, AUC ≈99.5% with Grad-CAM) [31]; Oliveira et al. on microinfarct/microhaemorrhage screening (100% WSI-level screening accuracy with multi-field of view (FOV) consensus and Grad-CAM [24]. In parallel, Kapasi et al. (high-throughput Alzheimer's quantification; r = 0.83–0.94 vs manual) and Corredor et al. (spatial immune-architecture in COVID-19 vs H1N1 lungs) provided rigorous quantitative readouts and internal reproducibility checks but did not present formal external/cross-site classifier validation, illustrating that, across the corpus, technical-stack descriptions are more common than fully standardised QA/QC or device-to-device robustness studies [23, 34].

## Discussion

Digitalization is transforming nearly every aspect of medicine, ushering in a new era of increased efficiency, accuracy, and accessibility in healthcare. From electronic health records and telemedicine to artificial intelligence and data analysis, digital tools are transforming the way healthcare providers work [35]. The adoption of digital technology for obtaining stained tissue sections on glass slides has significantly influenced the field of pathology [6].

DP reduces human subjectivity and introduces statistical rigor into histopathology, making findings more reproducible and reliable [36].

Despite its considerable potential, digital pathology (DP) has not yet been widely adopted in forensic practice, unlike its use in clinical pathology. A significant reason for this slower adoption is the high evidentiary standard: medico-legal applications require stricter validation, verifiability, and auditability, as well as a formal chain of custody that exceeds what is typically needed in research or routine diagnostics.

This review aimed to assess how DP is used in forensic settings and to identify the methodological improvements needed to make it routine. Although the existing literature is small, the included studies show that DP can be applied safely and effectively in forensics when appropriately governed: WSI-based primary review is feasible and concordant with glass; quantitative pipelines can stabilise measurements that matter in court; and AI-assisted tools can automate narrow, well-specified tasks (screening/triage) provided that reporting is transparent and traceable.

The College of American Pathologists (CAP) provides an evidence-based guideline for validating whole-slide imaging (WSI) before clinical use, as well as complementary accreditation checklists that incorporate these expectations into routine inspections [37, 38]. The 2021 Guideline Update recommends, for each intended application/use-case, a validation set of ≥ 60 cases, assessment of intra-observer concordance between glass and WSI separated by a washout period (≈2 weeks), and careful reconciliation of any discordant diagnoses; laboratories should be particularly concerned if overall WSI–glass concordance falls below ~ 95%. The guideline also stresses that validation must mirror the clinical environment (same workflow, monitors, network, and file formats), involve pathologists trained on the WSI system, and be repeated (revalidated) whenever a significant change occurs (e.g., scanner, software version, image compression, display hardware). These elements are operationalised in CAP's accreditation programme via discipline-specific checklists, which require documented validation plans and results, change control, user training/competency, and ongoing quality management for digital workflows.

Regarding applicability and current use, the most transparent case comes from a multicentre validation aligned with CAP principles. Pigaiani et al. demonstrated 97.8% diagnostic concordance between WSI and glass on 100 forensic cases, with excellent availability, following a blinded, washout, side-by-side design modelled on clinical validation playbooks [4]. This result is precisely the type of anchored evidence that makes WSI acceptable for everyday forensic histopathology, including second opinions, remote coverage, and retrospective case audits, as it proves that transitioning from glass to digital does not compromise the diagnostic endpoint.

Beyond primary review, quantitative DP adds objectivity where semi-quantitative grading is contested. In cardiovascular autopsies, D'Abbronzo et al. used WSI planimetry to produce a continuous % stenosis at the coronary level, which is repeatable, inspectable, and linked to the underlying image, thereby reducing ambiguity in borderline "sub-occlusion" scenarios [32]. In pulmonary fat embolism, Li et al. derived an H&E-only fat-area metric with a transparent threshold that separated fatal PFE from controls and mirrored reference methods (Oil Red O, Falzi) [30]. These are court-usable numbers, reproducible across readers and sessions, rather than purely narrative assessments.

Outside system-level validation, some studies show how WSI can provide relevant, quantitative endpoints. In this context, Benevento et al. digitized IHC-stained dura mater and used QuPath’s object-level cell detection to calculate positive-cell percentages and establish ROC-based thresholds that differentiate survival windows (minutes, hours, days) after TBI [33]. Two aspects are notable for forensic use: first, the metric is inherently auditable (tile-linked detections and reproducible thresholds); second, the diagnostic signal remains in mildly putrefied samples, suggesting that fibrous tissues, such as dura, may offer a decomposition-resistant substrate compared to brain tissue. Although the study is preliminary and needs multi-site replication and external validation, it demonstrates a practical pathway from marker “vitality” concepts to reliable, numeric outputs suitable for medico-legal reports.

AI-oriented works provided standard metrics (e.g., AUC, accuracy), but external validation was limited, a recurring constraint in emerging forensic datasets. The authors of the PFE and diatom papers explicitly highlighted sensitivity to acquisition parameters and the need for ongoing model maintenance [31, 31]. Where inter-observer agreement or reproducibility was assessed (e.g., Kapasi et al. rescan/magnification checks), results supported the stability of digital measures across conditions. Studies comparing digital assessments with conventional microscopy typically report high agreement for targeted questions (presence/extent of lesions), indicating that WSI-based review can reproduce key histological judgements in medico-legal contexts.

AI is already useful for triage of time-consuming reads when the target is narrow and visually well-defined. Zhou et al. matched expert diatom counts while reducing total processing time (∼6–7 h → ∼4 h) and exposing model attention with Grad-CAM, which is essential for traceability [31]. In post-mortem brain tissue, Oliveira et al. achieved 100% slide-level screening accuracy for microinfarcts/microhemorrhages by enforcing multi-field-of-view consensus and demonstrating scanner-agnostic behavior (Aperio + ZEISS) [24]. These pipelines do not replace expert diagnosis; they filter and prioritise, improving throughput without hiding the evidence trail.

So, why is forensic adoption slower than in clinical domains? First, validation expectations are higher: laboratories must show that WSI and any computational layer are fit for their specific forensic use. Second, chain-of-custody and auditability are non-negotiable: every measurement must be linkable to an immutable image state, with access logs, time stamps, and versioned analysis presets. Third, domain shift (stain variability, scanner parameters, tissue degradation) is common in medico-legal materials and can degrade model performance unless colour/stain management, as well as cross-device checks, are explicitly addressed. The included papers reflect this: they describe scanners and software in detail, and several documents repeat-scan, cross-magnification, or harmonisation steps, but only a minority report device-to-device robustness or external, cross-site testing, gaps that help explain the slower clinical translation in forensics.

Another concern regarding the use of DP in forensic science is data security and privacy, particularly when handling sensitive postmortem data [39]. The Digital Personal Data Protection Act (DPDPA) of 2023 in India mandates strict rules for collecting, storing, and processing personal data, including forensic evidence [40]. Adhering to these regulations is crucial to prevent unauthorized use, breaches, and the covert compromise of the integrity and security of personal forensic data. Modern digital pathology methods require the strongest encryption technology, access control systems, and data trail auditing to protect the confidentiality and integrity of forensic data. Additionally, cloud-based telepathology apps used in online consultations must comply with strict data-sharing regulations to prevent cross-border issues that could affect the legal admissibility of evidence [41, 42].

To support reproducibility, future forensic DP reports should include pixel size at scan**,** effective magnification**,** tile size/overlap, and, where quantification is central, repeatability metrics (intra-/inter-reader, rescan variability) with pre-specified acceptance limits. Reporting colour calibration and any stain normalisation method (or the decision to abstain) is likewise essential for downstream comparability. Widely used techniques include Macenko and Vahadane normalisation, which stabilise the appearance of stains across slides and scanners [43, 44].

Standardized naming conventions for slides and proper image storage durations are essential elements of digital pathology for traceability, interoperability, and regulatory compliance [39]. In accordance with the Digital Imaging and Communications in Medicine (DICOM) standards, Supplement 145, slide identifiers must conform to a standardized nomenclature to enable consistent data management and sharing across systems [45].

Upcoming prospects are therefore clear and immediate: implement CAP-style validation for each forensic application (pre-specified endpoints, washout designs, reconciliation of discordances, and re-validation after system changes), incorporate chain-of-custody by design (hashing/versioning of WSI and outputs, access logs, tile-level links for every number reported), include colour/stain governance and cross-device checks as standard sections in methods, and develop shared forensic benchmarks with separate external test sets for tasks like fat-area in PFE, diatom counts, injury morphometrics, and coronary % stenosis. These safeguards align with information security and digital evidence standards [46]. These steps collectively transform DP from a "promising technology" into a court-validated practice throughout the forensic workflow.

## Conclusion

The rise of digital pathology has reshaped clinical pathology, but its adoption in forensic practice remains sparse. In our review, only five studies directly applied digital pathology to forensic casework. However, where it has been utilized, digital pathology has proven to be safe and practical: whole-slide imaging offers glass-equivalent performance for primary review; digital workflows facilitate remote consultation and long-term archiving; and machine-learning–enabled software can assist with objective tasks like cell counting or lesion quantification.

To transition from promise to routine use in the medico-legal setting, four key actions are necessary. First, validate each forensic application by intended use according to CAP principles, including pre-specified endpoints, washout designs, reconciliation of discordances, and re-validation after material system changes. Second, integrate chain-of-custody into the workflow by design, with versioned images and outputs, timestamps, action logs, and direct links from every reported measurement to its visible region on the slide. Third, explicitly govern colour and staining, and include cross-device checks as a standard part of the Methods to identify and manage hidden drift. Fourth, develop shared forensic benchmarks with separate external test sets, enabling transparent performance comparison and broad generalization across sites and devices.

The successful implementation of digital pathology in forensic medicine will ultimately depend on close interdisciplinary collaboration among forensic pathologists, computer scientists and regulatory experts.

With these safeguards in place, digital pathology can advance from isolated demonstrations to dependable, court-proof practice that enhances quality, speed, and confidence in forensic conclusions.

## Keypoints

1. Digital pathology (DP) and whole-slide imaging (WSI) are emerging tools with promising applications in forensic medicine.
2. Our systematic review identified a growing number of studies addressing the use of DP for postmortem diagnostics, virtual autopsy and image archiving.
3. Implementation of DP in forensic workflows may enhance diagnostic accuracy, reproducibility and data sharing.
4. Interdisciplinary collaboration between forensic pathologists, IT specialists and regulatory experts is crucial for successful adoption.
